# Role of Mitochondria and Endoplasmic Reticulum in Taurine-Deficiency-Mediated Apoptosis

**DOI:** 10.3390/nu9080795

**Published:** 2017-07-25

**Authors:** Chian Ju Jong, Takashi Ito, Howard Prentice, Jang-Yen Wu, Stephen W. Schaffer

**Affiliations:** 1 Department of Pharmacology, College of Medicine, University of South Alabama, Mobile, AL 36688, USA; cjjong84@gmail.com; 2 Faculty of Biotechnology, Fukui Prefectural University, Fukui 910-1195, Japan; tito@fpu.ac.jp; 3 Program in Integrative Biology and Center for Complex Systems and Brain Sciences, College of Medicine, Florida Atlantic University, Boca Raton, FL 33431, USA; hprentic@health.fau.edu (H.P.); jwu@health.fau.edu (J.-Y.W.)

**Keywords:** oxidative stress, mitochondria, endoplasmic reticulum stress, apoptosis, caspase cascade, respiratory chain, mitochondria encoded proteins, tRNA^Leu(UUR)^

## Abstract

Taurine is a ubiquitous sulfur-containing amino acid found in high concentration in most tissues. Because of its involvement in fundamental physiological functions, such as regulating respiratory chain activity, modulating cation transport, controlling inflammation, altering protein phosphorylation and prolonging lifespan, taurine is an important nutrient whose deficiency leads to severe pathology and cell death. However, the mechanism by which taurine deficiency causes cell death is inadequately understood. Therefore, the present study examined the hypothesis that overproduction of reactive oxygen species (ROS) by complex I of the respiratory chain triggers mitochondria-dependent apoptosis in hearts of taurine transporter knockout (TauTKO) mice. In support of the hypothesis, a 60% decrease in mitochondrial taurine content of 3-month-old TauTKO hearts was observed, which was associated with diminished complex I activity and the onset of mitochondrial oxidative stress. Oxidative damage to stressed mitochondria led to activation of a caspase cascade, with stimulation of caspases 9 and 3 prevented by treatment of 3-month-old TauTKO mice with the mitochondria specific antioxidant, MitoTempo. In 12 month-old, but not 3-month-old, TauTKO hearts, caspase 12 activation contributes to cell death, revealing a pathological role for endoplasmic reticulum (ER) stress in taurine deficient, aging mice. Thus, taurine is a cytoprotective nutrient that ensures normal mitochondrial and ER function, which is important for the reduction of risk for apoptosis and premature death.

## 1. Introduction

Taurine is a β-amino acid found in very high concentration in excitable tissues. In certain species, such as the cat and fox, the amino acid is considered an essential nutrient, but in humans it is considered a semi-essential nutrient [1]. Neither humans nor cats readily synthesize taurine, therefore, the primary source of taurine for both species is the diet. For man, meat is a primary source of taurine, with the concentration of taurine being particularly high in seafood [2]. Hayes et al. [3] provided the first evidence that taurine is an essential nutrient for normal function of excitable tissues in cats, which developed a retinopathy when fed a taurine deficient diet. It was subsequently shown that taurine deficient cats also develop dilated cardiomyopathy [4]. In contrast to cats, adult rodents deprived of dietary taurine do not develop overt taurine deficiency, as they readily synthesize taurine in the liver [5]. Nonetheless, rodents become taurine deficient if exposed to a high concentration of a taurine transporter inhibitor or subjected to a genetic alteration that limits the uptake of taurine by excitable tissues. Hence, a taurine deficient cardiomyopathy develops in rodents lacking the myocardial taurine transporter (TauTKO mice) [6].

One of the primary physiological functions of taurine is its antioxidant activity, an action attributed to several mechanisms [7]. First, some investigators have attributed taurine’s antioxidant actions to elevations in the activity of antioxidant enzymes, however, by reducing the amount of damaging ROS (reactive oxygen species), taurine could indirectly elevate the activity of the antioxidant defenses. Second, taurine serves as an important anti-inflammatory agent, which involves a myeloperoxidase-catalyzed reaction between taurine and hypochlorous acid to generate an anti-inflammatory product, taurine chloramine. However, through the myeloperoxidase reaction, taurine also reduces the levels of the neutrophil-generated ROS, hypochlorous acid [8]. Third, reductions in intramitochondrial taurine content are associated with elevations in mitochondrial superoxide generation, leading to the suggestion that the mitochondria are the primary source of ROS generated by taurine deficient tissues [9]. While that conclusion may be valid for β-alanine-mediated taurine depletion, it is known that the taurine transport inhibitor, β-alanine, is a naturally occurring substance that exerts other actions within the cell besides reductions in taurine levels. Therefore, further studies were warranted to establish the source of mitochondrial ROS in the taurine deficient cell. Another unanswered question relates to the consequences of taurine deficiency-mediated oxidative stress. It is known that taurine exerts anti-apoptotic activity, however, it is unclear if its anti-apoptotic and antioxidant activities are directly related [10]. Moreover, it remains to be determined whether mitochondrial ROS is the only cause of apoptosis.

The taurine deficient heart is also characterized by diminished activity of the sarcoplasmic reticular (SR) Ca^2+^ ATPase [11], an effect consistent with the reduction in amplitude and prolongation of the relaxation phase of the Ca^2+^ transient, as well as the defect in systolic and diastolic function of the TauTKO heart. Alterations in Ca^2+^ homeostasis, as well as oxidative stress, have been known to also trigger endoplasmic reticulum (ER) stress and initiate an ER stress-mediated quality control process known as the unfolded protein response (UPR) [12]. Three transmembrane sensor proteins, PERK (protein kinase RNA (PKR)-like ER kinase), IRE-1 (inositol-requiring protein-1) and ATF6 (activating transcription factor 6) initiate distinct pathways of the UPR. In unstressed cells, the most abundant ER chaperone, GRP78, binds to PERK and ATF6, maintaining the chaperone in its inactive state. An elevation in unfolded proteins in the ER promotes the release of GRP78 from PERK and ATF6, allowing the two sensor proteins to initiate their respective UPR pathways. The aim of the UPR pathways is to reduce the cellular load of misfolded and unfolded proteins, restore ER function and allow the cell to function despite the unfolded protein load. However, if ER stress is excessive and overwhelms the capacity of the cell to restore normal ER function, two of the UPR pathways promote cell death. Hence, the present study examines the role of both mitochondrial ROS and ER stress in the initiation of apoptosis in TauTKO hearts.

## 2. Materials and Methods

### 2.1. Model of Taurine Deficiency (TauTKO)

Wild-type (WT) and homozygous taurine transporter knockout (TauTKO) mice were generated by mating heterozygous (TauTKO^+/−^) C57BL/6 mouse pairs [6]. This study was conducted using either 3- or 12-month-old mice. Animal handling and experimental procedures followed the Animal Welfare Act and the Guide for the Care and Use of Laboratory Animals and were approved by the Animal Care and Use Committee of the University of South Alabama.

### 2.2. Measurement of Mitochondrial Taurine Content

Mitochondria were prepared according the method described by Grishko et al. [13]. The mitochondrial fraction was re-suspended in mitochondrial buffer and a small aliquot was kept for protein concentration, while the remaining was used for taurine analysis. Isolated mitochondria were homogenized in ice-cold 1 M perchloric acid and 2 mM EDTA and subjected to centrifugation at 10,000× *g* for 10 min. The resulting supernatant was used to measure mitochondrial taurine content as determined by changes in absorbance at 355 nm [14].

### 2.3. Assay of Respiratory Chain Complexes

Complex I activity (NADH dehydrogenase) was evaluated according to the method of Ricci et al. [10]. Mitochondria, prepared according to the method of Grishko et al. [13], were suspended in 10 mM Tris buffer (pH 8.0) and then incubated for 5 min at 37 °C. The reaction was initiated by addition of NADH and was monitored at 340 nm for 3 min, after which 5 μM rotenone was added and changes in absorbance at 340 nm were observed for an additional 2 min. Complex I activity was calculated from the difference between NADH oxidation in the presence and absence of rotenone and expressed as mmol/min/mg protein.

Complex II activity (succinate dehydrogenase) was determined according to the method of Ricci et al. [10]. Isolated mitochondria were suspended in 50 mM potassium phosphate buffer (pH 7.4) containing 20 mM succinate and incubated for 3 min at 37 °C. To the mitochondrial suspension was added 50 mM potassium phosphate buffer containing 500 μM 2,6-dichlorophenolindophenol, 20 mM KCN, 20 μg/mL rotenone and 20 μg/mL antimycin A. The reaction was initiated by addition of 25 μM decylubiquinone and followed for 3 min. Complex II activity (succinate dehydrogenase) was determined from the reduction of 2,6-dichlorophenolindophenol at 600 nm and expressed as μmol/min/mg protein [10].

Complex III was determined using the method of Chen et al. [15]. Isolated mitochondria were suspended in 50 mM Tris buffer (pH 7.4) containing 250 mM sucrose, 1 mM EDTA, 50 μM oxidized cytochrome c, 2 mM KCN and 10 μg/mL rotenone. After 10 min pre-incubation at 37 °C, the reaction was initiated by addition of 10 mM decylubiquinol and monitored at 550 nm for 3 min before addition of 40 μM antimycin, after which the reaction was further monitored for 2 min. Complex III activity (expressed as mmol/min/mg protein) was calculated from the difference in cytochrome c reduction in the presence and absence of antimycin A.

Complex IV was assayed using the method of Ma et al. [16]. Mitochondria were suspended in 10 mM potassium phosphate buffer (pH 7.4). The reaction was initiated by addition of substrate, ferrocytochrome c (50 μM), and monitored at 550nm. Complex IV activity was evaluated from the rate of reduced cytochrome c oxidation in the presence and absence of KCN (2 mM).

Complex V was assayed as described previously by our group [17]. The assay is based on a spectrophotometric assay of ATP combining pyruvate kinase and lactate dehydrogenase and monitoring changes in NADH oxidation at 340 nm. Complex V activity was evaluated from the rate of NADH oxidation in the presence and absence of oligomycin (1 μg/mL).

### 2.4. Quantitative Real Time PCR Method of Measuring Levels of ND6 mRNA

Total RNA was isolated from the heart using TRIzol LS Reagent (Invitrogen, Carlsbad, CA, USA) according to the protocol. Quantitative real time PCR was then performed using the iScript One-Step RT-PCR Kit with SYBR Green (Bio-Rad, Hercules, CA, USA) according to the protocol. Primer sequences for ND6 were 5′-ataggatcctcccgaatcaaccct-3′ (forward) and 5′-aggattggtgctgtgggtgaaaga-3′ (reverse), and primer sequences used for β-actin were 5′-gtgacgttgacatccgtaaa-3′ (forward) and 5′-ctcaggaggagcaatgatct-3′ (reverse).

### 2.5. Assay of Aconitase Activity

The mitochondrial fraction was re-suspended in buffer containing 50 mM Tris-HCl, pH 7.4 and 0.2 mM sodium citrate. Aconitase was assayed using the Bioxytech Aconitase-340 kit by monitoring the increase in NADH absorbance at 340 nm. Aconitase activity was normalized relative to succinate dehydrogenase (complex II activity), whose activity is unaffected by oxidative stress.

### 2.6. Determination of Glutathione Redox State

Hearts were homogenized in ice-cold 50 mM phosphate buffer pH 7.4 and homogenates were then centrifuged at 10,000× *g* for 10 min. A small aliquot of the supernatant, which is defined as the total lysate, was kept for protein concentration and the remaining was deproteinized with 1 M perchloric acid and 2 mM EDTA and subjected to centrifugation at 10,000× *g* for 10 min. The supernatant was neutralized to pH 6–7 and centrifuged again at 10,000× *g* for 10 min. The resulting supernatant was used to assess the glutathione redox state using a glutathione assay kit. The glutathione redox state was determined as the ratio of reduced glutathione (GSH) to oxidized glutathione (GSSG). Both GSH and GSSG content were determined as an increase in absorbance at 405 nm. GSSG content was measured after derivatizing pre-existing GSH in each sample with 2-vinylpyridine.

### 2.7. Measurement of Protein Carbonylation

Total lysates were prepared by initially homogenizing hearts in radioimmuno-precipitation assay (RIPA) lysis buffer. The homogenates were centrifuged at 10,000× *g* for 20 min and the supernatant was finally collected. The protein concentration was measured by BCA assay. Protein (10–20 µg) was assayed for the degree of protein carbonylation using the Oxyblot Protein Oxidation Detection kit (Catalog No S7150, Millipore; Darmstadt, Germany). Briefly, proteins were derivatized with 2,4-dinitrophenylhydrazine (DNPH), separated by polyacrylamide gel electrophoresis and subjected to Western blotting. Carbonylated proteins were detected utilizing the primary antibody, which is specific for the DNP moiety of proteins.

### 2.8. Determination of Protein Content via Western Blot Analysis

Total lysates were prepared by homogenizing hearts in RIPA lysis buffer (50 mM Tris base, pH 8.0, 150 mM NaCl, 0.5% deoxycholic acid, 1% NP-40, 0.1% sodium dodecyl sulfate). Homogenates were centrifuged at 10,000× *g* for 10 min and the resulting supernatants were collected as total lysates. Isolated mitochondria were prepared as described earlier. The mitochondrial fraction was suspended in RIPA lysis buffer. The protein concentration was measured by the bicinchoninic assay (BCA). Protein (20–30 µg) was mixed with an equal volume of 5× sample buffer (1.25 mM Tris HCl, pH 6.8, 1% sodium dodecyl sulfate, 10% glycerol, 5% β-mercaptoethanol) and then boiled for 5 min. Proteins were separated by sodium dodecyl sulfate polyacrylamide gel electrophoresis (SDS-PAGE) and transferred onto a nitrocellulose membrane. The membranes then were blocked in blocking buffer (5% milk in tris buffered saline with Tween 20) and incubated with an appropriate primary antibody overnight at 4 °C. The primary antibodies used were specific for the following proteins: ND1 (sc-20493), ND2 (sc-20496), ND3 (sc-26760), ND4 (sc-20499), ND6 (Molecular Probes A31587), COX1 (Molecular Probes A6403), cytochrome b (sc-11436), SDH (sc-25851), β-actin (sc-130656) caspase 3 (sc-7148), caspase 12 (Cell Signaling #2202), caspase 9 (Cell Signaling #9508), GRP78 (sc-13968), CHOP (Cell Signaling #2895S), phospho-IRE1 (Abcam #48187), PERK (sc-13073), phosphor-PERK (sc-32577), ATF4 (Cell Signaling #11815), XBP-1 (Abcam #37152). PARP (sc-7150). The next day, membranes were washed before being incubated with an appropriate secondary antibody. After washing, western blots were analyzed by enhanced chemiluminescent reagents.

### 2.9. Evaluation of Mitochondrial Oxidative Stress

WT and TauTKO mice were administered either PBS (vehicle) or MitoTempo (1.4 mg/kg/day) by intraperitoneal injection for 7 consecutive days. On the 7th day, mice were killed and hearts were immediately removed and rapidly frozen with aluminum tongs cooled in liquid nitrogen. Samples were stored at −80 °C.

### 2.10. Statistical Analyses

All results were reported as means ± SEM. The statistical significance of the data was determined using the Student’s *t*-test for comparison within groups or ANOVA followed by the Newman-Keuls test for comparison between groups. Values of *p* < 0.05 were considered statistically significant.

## 3. Results

### 3.1. Effect of Taurine on Mitochondrial Function

**Figure 1:**
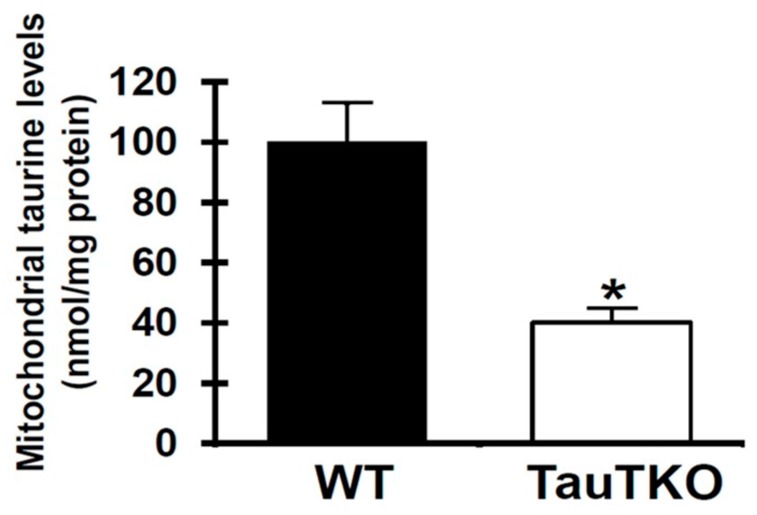
Reduced mitochondrial taurine content of taurine transporter (TauTKO) hearts. Following isolation of the mitochondrial fraction from homogenized hearts, extracts were prepared by precipitating protein. Mitochondrial taurine content was then measured. Values shown represent means ± SEM of 5–7 different hearts. * *p* < 0.05. WT, the wild-type.

Ito et al. [6] have shown that global deletion of the taurine transporter gene suppresses taurine uptake by the heart, as shown by the presence of nondetectable levels of taurine in the cytosol of the TauTKO heart vs. nearly 28 mM in the wild-type (WT) heart. However, the mitochondria of the 3-month-old TauTKO heart appear to be resistant to the loss of taurine, as mitochondrial taurine content of the TauTKO heart was 40 nmol/mg protein, which is only 60% less than the taurine content of the age-matched, WT heart (Figure 1).

**Figure 2:**
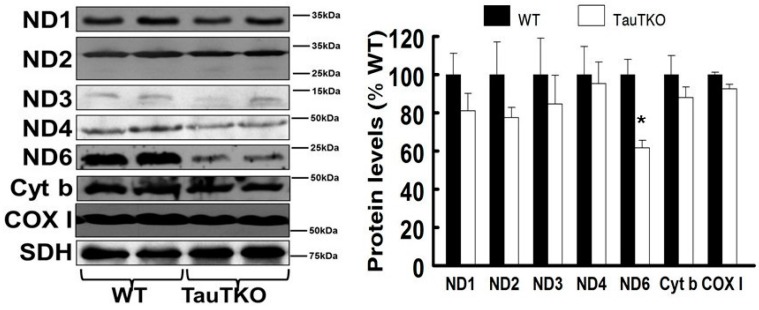
ND6 is reduced in TauTKO hearts. The mitochondrial fraction was isolated from homogenized hearts and then subjected to western blot analyses. The left panel shows representative gels for ND1, ND2, ND3, ND4, ND6, Cyt b (cytochrome b) and COX I (cytochrome c oxidase I), with succinate dehydrogenase (SDH) serving as the loading control. The right panel shows the means ± SEM of the mitochondrial protein/SDH ratio of 6–9 different hearts. Values are expressed relative to wild-type (WT), where WT is fixed at 100%. * *p* < 0.05.

It has been previously shown that formation of the mitochondrial taurine conjugate, 5-taurinomethyluridine-tRNA^Leu(UUR)^, enhances UUG decoding [18]. Of the three leucine codons (CUN, UUA, UUG) present in the mitochondria, two (UUA and UUG) interact with the anticodon of tRNA^Leu(UUR)^ during the course of protein synthesis. However, the interaction of UUG is highly dependent on the presence of the 5-taurinomethyluridine conjugate of tRNA^Leu(UUR)^ [19,20]. The most frequently used leucine codon in mitochondrial protein biosynthesis is CUN, with UUA being intermediate and UUG being the least used codon [20]. Because of the importance of the taurine conjugate in the interaction of the UUG codon with the anticodon of tRNA^Leu(UUR)^, we tested the hypothesis that taurine deficiency selectively diminishes the expression of UUG-dependent, mitochondria encoded proteins in the heart. As seen in Figure 2, the level of ND6 was significantly reduced in the TauTKO heart relative to that of several mitochondria encoded proteins examined in the WT heart.

To provide insight into the mechanism underlying the decline in ND6 protein content, the mRNA level of ND6 was determined in TauTKO and WT hearts. In contrast to most conditions in which protein expression is altered by taurine deficiency, no significant change in the mRNA content of ND6 was observed (data not shown; *p* = 0.15).

**Figure 3:**
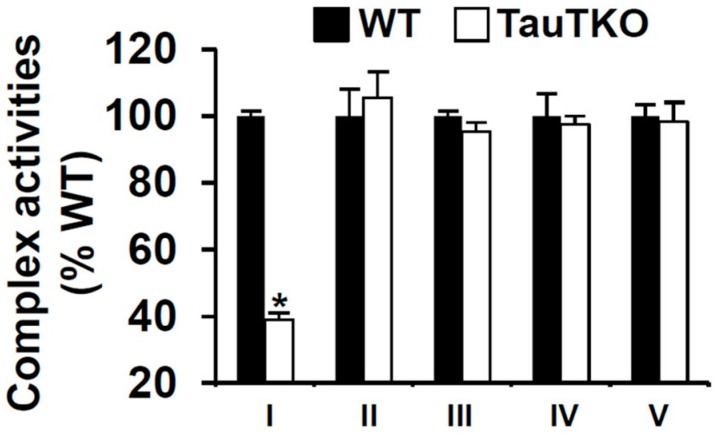
Taurine depletion decreases complex I activity. Isolated mitochondrial fractions of WT and TauTKO hearts were assayed for the activities of complexes I–V. Values of individual complexes shown represent means ± SEM of 4–6 different hearts. * *p* < 0.05.

ND6 is not only a subunit of complex I but a facilitator of complex I assembly [20], therefore, we tested whether taurine deficiency specifically decreases the activity of complex I without affecting the activities of the other respiratory chain complexes. In accordance with that hypothesis, we found that the activity of complex I was reduced 60% in the mitochondria of the TauTKO heart while the activities of complexes II–V were unaffected by taurine deficiency (Figure 3).

**Figure 4:**
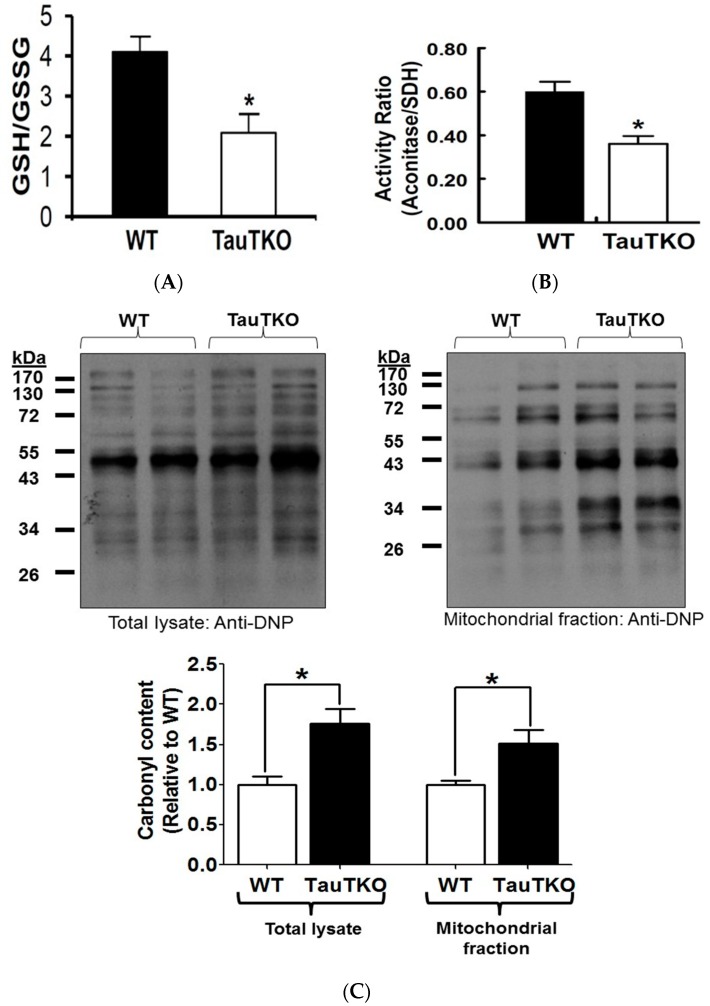
Taurine depletion causes oxidative stress. (**A**) Reduced (GSH) and oxidized (GSSG) glutathione content were determined and the data expressed as the glutathione redox state (GSH/GSSG), with values shown representing means ± SEM of 6–8 hearts. * *p* < 0.05; (**B**) Aconitase activity of WT and TauTKO mitochondria were assayed and normalized relative to SD activity. Values represent means ± SEM of 4–6 hearts. * *p* < 0.05; (**C**) Following preparation of total heart lysates and the mitochondrial fraction, proteins were derivatized with 2,4-dinitrophenylhydrazine and then subjected to western blot analysis of carbonylated proteins. The top panels show representative gels of carbonylated proteins of total lysate and the mitochondrial fraction. Values shown in the bottom panel represent means ± SEM for relative cellular and mitochondrial carbonylated protein content from 4–5 hearts. Values are expressed relative to WT, where WT is fixed at 1.0. * *p* < 0.05.

Reduced flux of electrons through the respiratory chain is often associated with the diversion of electrons from the respiratory chain to the acceptor, oxygen, forming in the process superoxide anion. To determine if the mitochondria of the TauTKO heart are oxidatively stressed, the glutathione redox ratio (GSH/GSSG) of TauTKO hearts was determined. Supporting the view that the taurine deficient heart is oxidatively stressed, it was shown that the glutathione redox ratio was 2-fold less in the 3-month-old TauTKO heart than in that of the age-matched, WT heart (Figure 4A). However, the levels of ROS differ between various organelles within the cell, with mitochondria being unique because of their ability to not only generate superoxide but also to degrade ROS [21]. Therefore, to determine if taurine deficiency enhances oxidative stress in the mitochondria, the activity of the ROS-sensitive, citric acid cycle enzyme, aconitase, was assayed. As seen in Figure 4B, taurine deficiency is associated with a 30% decrease in aconitase activity. Another marker of oxidative stress is protein carbonylation, in which amino acid residues, such as lysine, arginine, proline and threonine, undergo oxidation to form a protein carbonyl derivative containing an aldehyde or carbonyl group [22]. Figure 4C shows that the amount of carbonylated protein in the cellular lysate and mitochondrial extract of the taurine deficient heart increased 50%. Together, these data show that taurine deficiency is associated with an increase in oxidative stress.

### 3.2. Taurine Depletion Induces Cell Death—An Effect Mediated by Oxidative Stress

**Figure 5:**
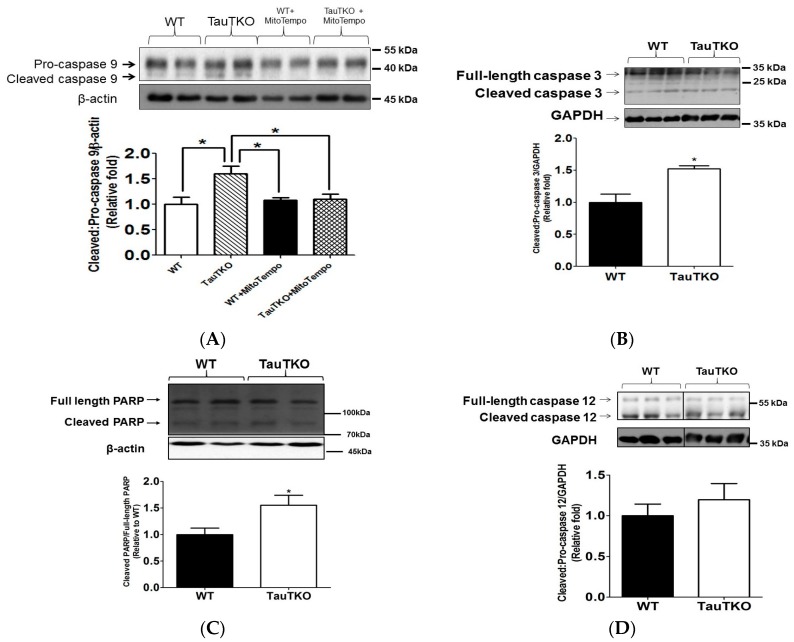

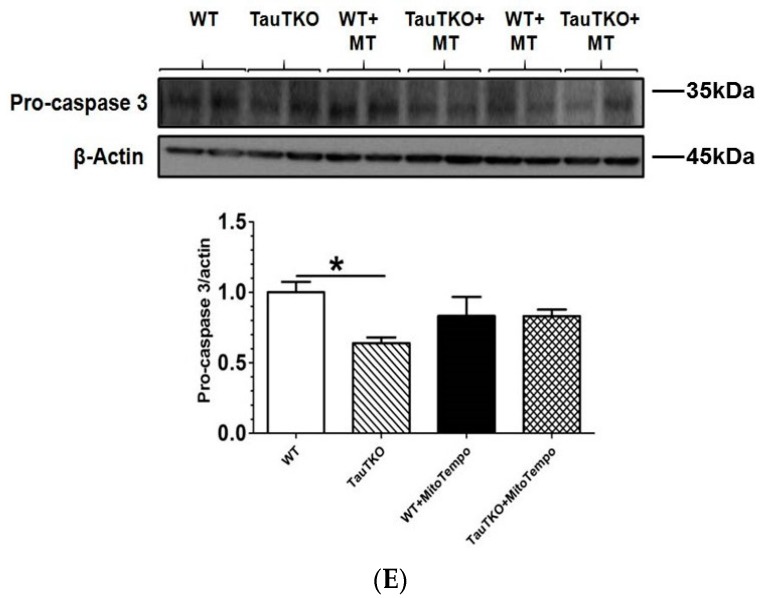
Taurine depletion induces apoptosis. Total heart lysates of 3-month-old TauTKO and WT hearts were subjected to western blot analyses of the active forms of caspase 9, caspase 3 and caspase 12, as well as the inactive pro-caspase forms of the three proteases (**A**–**E**). In (**A**), the data are expressed as the ratio of active caspase 9/pro-caspase 9 while in (**B**), the data are depicted as the ratio of cleaved caspase 3/pro-caspase 3. In (**C**), the data are expressed as cleaved PARP levels. In (**D**), the ratio of cleaved/pro-caspase 12 is shown. In (**E**), TauTKO and WT mice were treated with the mitochondria-specific antioxidant, MitoTempo, for 7 days and changes in the levels of the inactive pro-caspase 3 zymogen were determined. Each panel contains a representative gel. The representative bands for WT and TauTKO were spliced from one original gel and the splice junction is indicated by the black splicing line. Values shown represent means ± SEM of 6–9 hearts. All values are expressed relative to WT, where WT is fixed at 1.0. * *p* < 0.05.

Excessive oxidative stress commonly triggers mitochondria-dependent cell death via activation of the initiator protease, caspase 9. As shown in Figure 5A, there is a significant increase in the levels of the active, cleaved form of caspase 9 in the 3-month-old TauTKO heart, which resulted in a net increase in the active cleaved/pro-caspase 9 ratio of 60% relative to that of the WT heart (Figure 5A). To determine if mitochondrial oxidative stress contributes to the activation of caspase 9, the cleaved/pro-caspase 9 ratio was examined in 3-month-old TauTKO mice that had been administered MitoTempo (1.4 mg/kg/day) by intraperitoneal injection for 7 consecutive days before removal of the heart. Although treatment with MitoTempo had no effect on the generation of the active form of caspase 9 in the WT heart, it abolished the activation of caspase 9 in the 3-month-old TauTKO heart. 

The active form of caspase 9 is a mitochondrial-localized protease, which initiates an apoptotic cascade by cleaving pro-caspase 3 to its active, cleaved caspase 3 form. Figure 5B shows that the content of the active, cleaved form of caspase 3 is increased in 3-month-old TauTKO mice relative to that of the age-matched control WT mice, causing the ratio of cleaved caspase 3/pro-caspase 3 to increase 50% in young TauTKO hearts relative to that of young WT hearts. Activation of caspase 3 subsequently cleaves poly-ADP (ribose) polymerase (PARP) and induces apoptosis. Indeed, as shown in Figure 5C, a significant 50% increase in the cleaved levels of PARP is observed in TauTKO hearts. In contrast to caspases 3 and 9, the level of the cleaved, active form of caspase 12, which is activated following initiation of UPR by ER stress, was identical in the 3 month- old TauTKO and WT hearts (Figure 5D). Treatment of 3-month-old TauTKO mice with MitoTempo abolishes the significant decrease in pro-caspase 3 content but the mitochondrial antioxidant has no effect on pro-caspase 3 levels in WT mice (Figure 5E).

### 3.3. Potential Crosstalk between Mitochondria and ER

**Figure 6:**
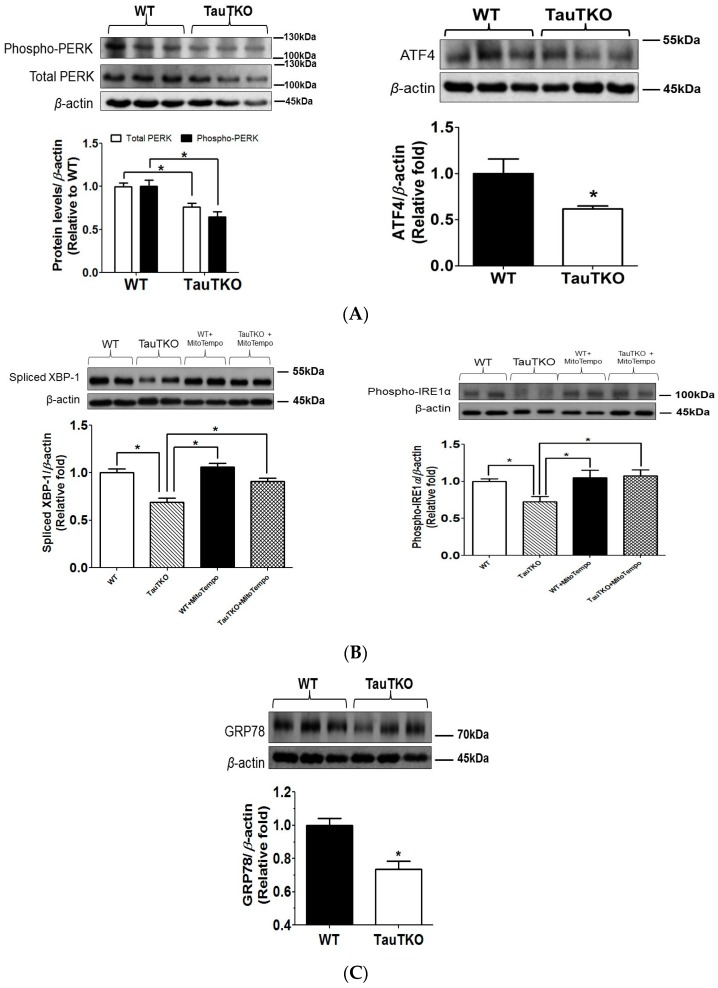
Unfolded protein response (UPR) is not activated in TauTKO hearts at an early age. Total lysates from 3-month-old WT and TauTKO mice treated with or without MitoTempo were subjected to western blot analysis of (**A**) phosphorylated PERK and ATF4 (**B**) spliced XBP-1 and phosphorylated-IRE1 and spliced XBP-1 and (**C**) GRP78. Each panel contains a representative gel and summation data expressed as means ± SEM of 6–9 hearts. Values are expressed relative to WT, where WT is fixed at 1.0. * *p* < 0.05.

Crosstalk between mitochondria and the sarcoplasmic reticulum of the heart has been observed in specific mitofusion 2-containing mitochondrial-reticular microdomains [23]. It has been demonstrated that ROS and Ca^2+^ are capable of promoting crosstalk between the two organelles [24,25]. According to Ramila et al. [11], Ca^2+^ handling by the ER (SR) of the TauTKO heart is defective, a change attributed to impaired activity of several protein kinases and a phosphatase. Because high levels of Ca^2+^ are required for normal protein folding by the ER [24], the size of the ER stores and the activity of the SR Ca^2+^ ATPase are determinants of ER stress [26]. Moreover, ROS perturbs the ER, thereby initiating ER stress, in part by disrupting proper disulfide-sulfhydryl interchange and disulfide bridge formation via the oxidoreductases that require an oxidative environment in the ER lumen to promote proper protein folding and ER function [27]. Because of the link between mitochondria and the ER, as well as the importance of ER stress in the initiation of apoptosis, we examined the status of two of the three transmembrane sensor proteins (PERK and IRE1) and their downstream effectors; the two UPR pathways examined are involved in the initiation of apoptosis [28]. As shown in Figure 6A, the content of phosphorylated PERK and ATF4, which are components and biomarkers of the PERK pathway, are reduced 25–35% in the 3-month-old TauTKO heart relative to that of the WT control heart. Similarly, two biomarkers of the IRE1 signaling pathway, phospho-IRE1 and spliced XBP-1, were 20% lower in the 3-month-old TauTKO heart than in the 3-month-old WT heart, an effect that was diminished upon treatment of the animal with MitoTempo (Figure 6B). Common to both the PERK and IRE1 pathways is the chaperone, GRP78, which in normal ER is associated with the transmembrane sensor proteins, maintaining them in an inactive state. A key beginning event in the initiation of UPR is the dissociation of GRP78 from the three transmembrane sensor proteins, freeing them to activate distinct UPR pathways. In most examples of ER stress and UPR signaling, GRP78 is upregulated and UPR signaling is stimulated [29,30]. However, as seen in Figure 6C, levels of GRP78 are reduced in the 3-month-old TauTKO heart, an effect associated with inhibition of UPR signaling. The pattern that develops in the 3-month-old TauTKO heart is the mirror image of that seen normally during initiation of ER stress and activation of UPR signaling, suggesting that UPR is not initiated in the young TauTKO heart.

**Figure 7:**
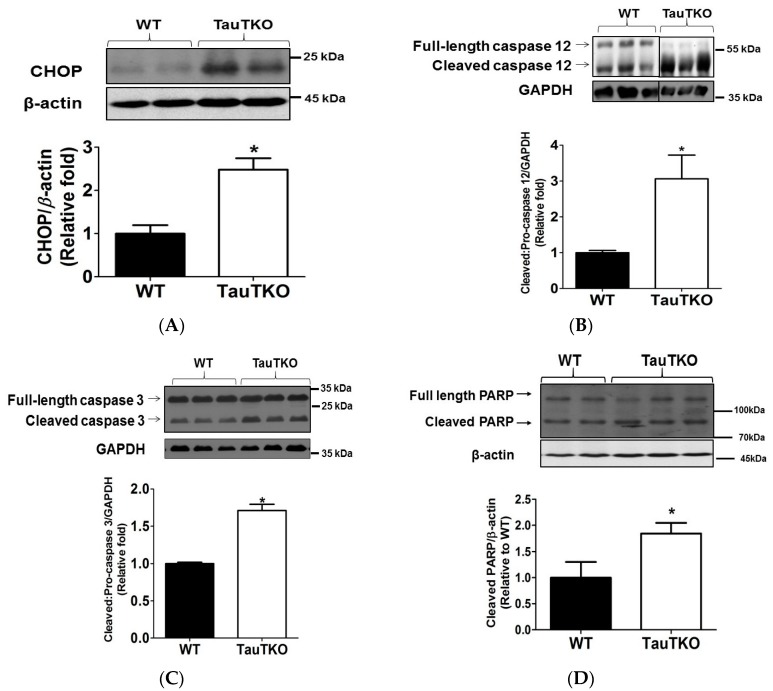
CHOP and UPR caspase cascade are activated in older TauTKO hearts. Total heart lysates from 12-month-old TauTKO and WT mouse hearts were subjected to western blot analysis of (**A**) CHOP, (**B**) caspase 12, (**C**) caspase 3 and (**D**) PARP. Each panel contains a representative gel and summation data expressed as means ± SEM of 6–9 hearts. The representative bands for WT and TauTKO shown in B were spliced from one original gel and the splice junction is indicated by the black splicing line. All values are expressed relative to WT, where WT is fixed at 1.0. * *p* < 0.05.

To further test the role of UPR in taurine deficiency-mediated apoptosis, the content of the two most important mediators of UPR-mediated apoptosis, CHOP (CCAAT/enhancer binding protein), and the ER localized protease, caspase 12, were determined. Figure 7A shows that CHOP content was significantly elevated in the hearts of the 12-month-old TauTKO mice relative to those of those of year-old WT hearts. Similarly, levels of the active, cleaved form of caspase 12, which is likely activated by calpain and the caspase cascade [31], are nearly 3.0 fold greater in the TauTKO heart than in that of the WT heart (Figure 7B).

Following its cleavage, caspase 12 is known to activate caspase 3, an effector protease that induces downstream apoptotic events. In the 3-month old TauTKO mouse heart, the activation of caspase 3 is solely related to caspase 9 cleavage. However, by the time the TauTKO mouse reaches one-year of age, caspase 12 is also capable of activating caspase 3. As seen in Figure 7C, there is a 70% increase in the cleaved/pro-caspase 3 ratio and a significant 70% increase in the cleaved PARP. Because caspases 9 and 12 are more active in the 1 year-old TauTKO mouse than the age-matched WT mouse, we assume that the active state of caspase 3 in the 1 year-old TauTKO is related to the activation of both initiator caspases.

Activation of caspases 9 and 12 cause cardiomyocyte death, which is an important process in the development of cardiomyopathy [6]. In the taurine deficient cat, onset of cardiomyopathy can ultimately be fatal. Therefore, we examined the mortality rates of TauTKO and WT mice over the time period of 3 months-of-age and 12-months-of-age. Beginning with 71, 3-month-old WT mice and 59, 3-month-old TauTKO mice, we found that one WT mouse died between the ages of 3-months and 1 year, which corresponds to a mortality rate over the designated time frame of 1.4%. By comparison, the mortality rate of the TauTKO mice was 3.4%. As the animals aged, the mortality rate increased, particularly in the TauTKO mice [32]. Therefore, the increase in myocardial apoptosis is associated with an elevation in the mortality rate of the taurine deficient animal.

## 4. Discussion

**Figure 8:**
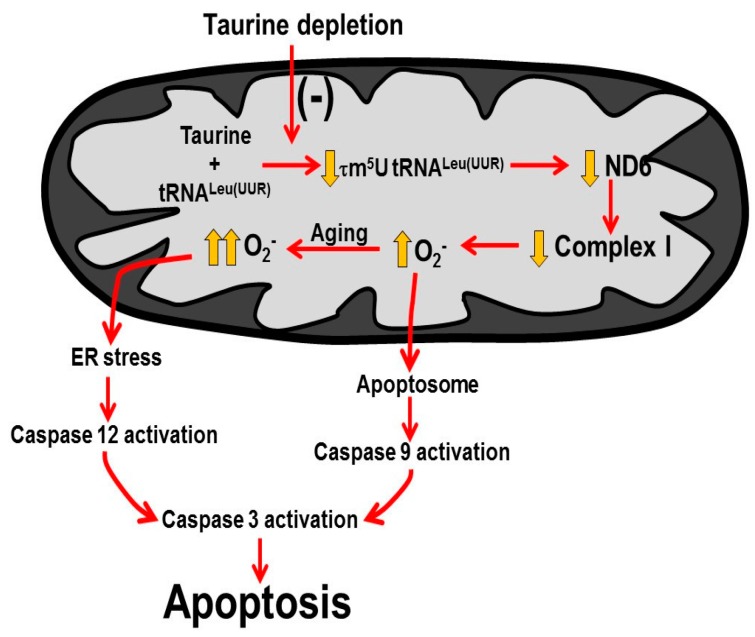
Mechanisms underlying taurine deficiency-mediated myocardial cell death.

The present study demonstrates that myocardial deficiency of the natural nutrient, taurine, leads to impaired mitochondrial complex I activity, a defect associated with an increase in mitochondrial oxidative stress and the initiation of mitochondria-mediated apoptosis as early as 3-months-of-age (Figure 8). However, by 12-months-of-age, the ER-localized protease, caspase 12, becomes active and contributes to the initiation of the caspase cascade and the increase in mortality rate (Figure 8).

### 4.1. Mitochondrial Actions of Taurine

The animal model utilized in the present study is the taurine transporter knockout mouse (TauTKO), whose cytosolic taurine levels in the heart are too low to detect [6]. By comparison, the mitochondria from 3-month-old TauTKO hearts contain 40% of normal taurine content (Figure 1). Despite the resistance of the mitochondria to taurine loss, the 60% decline in taurine content noted in the present study is associated with significant respiratory chain dysfunction (Figure 2 and Figure 3).

In normal mitochondria, taurine forms a conjugate with a uridine residue located in the anticodon wobble position of mitochondrial tRNA^Leu(UUR)^ [18,20]. This modification strengthens the interaction of the AAU anticodon of tRNA^Leu(UUR)^ with the UUG codon of mitochondrial mRNAs, thereby facilitating UUG decoding and the translation of mitochondria encoded proteins whose mRNA contains multiple UUG codons [19,20]. The present study shows that one of the mitochondria encoded proteins, ND6, is highly sensitive to taurine deficiency, likely related to the high number of UUG codons (8) found in its mRNA and the large number of leucine residues of ND6 that are dependent on the UUG codon (42%) [20,33,34].

The reduction in ND6 levels in the TauTKO heart is not caused by a decrease in ND6 mRNA. Rather, the reduction in ND6 expression appears related to a decrease in 5-taurinomethyluridine-tRNA^Leu(UUR)^ levels, thereby diminishing the interaction between the UUG codons of ND6 mRNA and the AAU* anticodon of tRNA^Leu(UUR)^, where U* represents the modified wobble uridine moiety, 5-taurinomethyluridine and U represents unmodified uridine. Because the mRNA of ND6 contains 8 UUG codons, the weak interaction between the codon UUG and anticodon AAU, would lead to a decrease in ND6 biosynthesis.

ND6 serves an important function in complex I, as it is not only a structural subunit of the complex but also facilitates proper assembly of the complex [35]. Therefore, taurine deficiency is associated with significant decreases in complex I activity and mitochondrial respiration, the latter defect bypassed in taurine deficient mitochondria respiring a complex II substrate, such as succinate [9,20,33].

The bottleneck that develops in complex I of the taurine deficient heart has a dramatic effect on energy metabolism [36]. Not only is flux of electrons through the respiratory chain diminished by impaired NADH dehydrogenase activity (innate complex I enzyme activity), but the increase in the myocardial NADH/NAD^+^ ratio (resulting from the decrease in NADH dehydrogenase activity) adversely affects energy metabolism by inhibiting key enzymes of glucose and fatty acid metabolism [36]. Thus, the taurine deficient heart becomes energy deficient, an effect that likely contributes to impaired mechanical performance of the heart.

The decline in respiratory chain electron activity, as noted for taurine deficiency, respiratory chain inhibitors, mitochondrial damage and respiratory chain mutations, is associated with enhanced superoxide generation at one of two respiratory chain sites, complex I or complex III [21,37,38]. Since taurine deficiency specifically decreases complex I activity, it is not surprising that it functions like the complex I inhibitor, rotenone, which stimulates the generation of superoxide anion by complex I [9,21]. Other factors that are capable of increasing the generation of superoxide by complex I are increased proton-motive force, reduced CoQ, elevated NADH/NAD^+^ ratio and increased oxygen concentration [39]. Thus, the most important properties increasing ROS generation by the taurine deficient heart are diminished electron flow and elevated NADH/NAD^+^ ratio [36].

Superoxide and other ROS generated by complex I are targeted to the mitochondrial matrix, while complex III preferentially targets ROS to the intermembrane space [40]. Thus, the proteins undergoing oxidation by complex I-generated ROS are different from those oxidized by complex III-generated ROS. One of the proteins modified in the taurine deficient heart is aconitase, an enzyme from the citric acid cycle that is located in the matrix. Because aconitase is abundant in the mitochondrial matrix, the observed decrease in activity is unlikely to alter citric acid cycle flux, which is largely diminished in the TauTKO heart because of elevations in the NADH/NAD ratio [36].

On the other hand, the oxidation of specific mitochondrial proteins can lead to mitochondrial outer membrane permeabilization (MOMP) and the release of cytochrome c from the mitochondria. Normally, most cytochrome c is associated with the inner mitochondrial membrane through weak electrostatic interactions with acidic phospholipids although a stronger interaction develops between cytochrome c and the phospholipid, cardiolipin [41]. ROS generated by complexes I and III are capable of oxidizing cardiolipin, breaking the interaction with cytochrome c. Therefore, massive amounts of cytochrome c become available for release from the mitochondria upon MOMP. The prominent mechanism for initiation of MOMP is oligomerization of pro-apoptotic Bcl-2 family members, Bax and Bak. However, MOMP can also be initiated at the level of the mitochondrial inner membrane upon formation of the mitochondrial permeability transition pore, which allows the transfer of solutes with a molecular weight of 1500 Da or less. Massive matrix swelling can cause MOM rupture, releasing cytochrome c [42]. In the cytosol, cytochrome c combines with ATP and APAF-1 to form a large complex, referred to as the apoptosome. According to Hu et al. [43], activation of the zymogen of caspase 9 by the apoptosome leads to a 2–3 order of magnitude increase in proteolytic activity of caspase 9. In turn, the active form of caspase 9 enhances the proteolytic activity of the effector, caspase 3. Several lines of evidence indicate that this sequence of events is responsible for the death of cardiomyocytes in the 3-month-old taurine deficient heart. First, the increase in ROS in taurine deficient cells is restricted to the mitochondria [9,33]. Second, treatment of TauTKO mice with the mitochondria-specific antioxidant, MitoTempo, diminishes the rise in mitochondrial ROS and prevents the activation of caspases 9 and 3. Third, β-alanine-mediated taurine deficiency leads to mitochondrial fragmentation and oxidative stress, effects reversed by restoration of normal taurine content [9]. Fourth, Montessuit et al. [44] reported that mitochondrial fragmentation facilitates Bax oligomerization and apoptosis. Although it was suggested that the factor that mediates mitochondrial fragmentation, Drp1, may directly participate in Bax oligomerization, Kashnareva et al. [45] found no Drp1 in MOM, ruling out Drp1 in MOMP. Recently, Shetewy et al. [9] showed that taurine deficient cells undergo mitochondrial fragmentation, which may increase the susceptibility of taurine deficient cells to Bax-induced oligomerization and MOMP. Further study of this hypothesis is warranted. Fifth, hearts of TauTKO mice, but not those of WT mice, undergo cleavage of the natural caspase 3 substrate, PARP.

### 4.2. Role of ER Stress in TauTKO Hearts

The 3-month-old TauTKO mouse exhibits no evidence of ER stress and UPR signaling despite severe mitochondrial oxidative stress, diminished energy metabolism, accumulation of ubiquitinated and carbonylated proteins and even impaired Ca^2+^ handling by the SR [11,33,36,46]. The reason for the lack of UPR signaling in 3-month-old taurine deficient mice is unclear but it may be related to the low levels of GRP78, which are below the threshold required to activate the protein sensors. Nonetheless, the findings clearly show that the oxidant status of the mitochondria influences ER function and that the TauTKO heart does not develop overt ER stress until the animal is older. As expected, downstream pro-apoptotic effectors, CHOP and caspase 12, remain within the normal range in the 3-month-old TauTKO heart.

We have previously shown that UPR signaling is enhanced in skeletal muscle of older TauTKO mice [32]. Although the status of caspase 12 and CHOP was not examined in our earlier study, we did observe an increase in GRP78 levels, as well as enhanced ATF6 and IRE1 signaling (ATF6 and spliced XBP1 content). Since the activation of caspase 12 is associated with IRE1 signaling, the TauTKO heart appears to be well equipped to activate caspase 12. In agreement with that notion, we found that caspase 12, as well as CHOP, are upregulated in the 12-month-old TauTKO heart.

It has previously been shown that taurine treatment downregulates CHOP and decreases caspase 12 activity in models of severe stress, thereby diminishing cell death [47,48,49]. In the case of glutamate excitotoxicity and stroke, taurine treatment suppresses ATF6 and IRE1 signaling [49], the same pathways affected by taurine deficiency. Moreover, both taurine treatment and taurine deficiency alter similar biochemical processes, Ca^2+^ transport and oxidative stress [11,33,49]. Thus, taurine treatment and taurine deficiency appear to affect caspase 12 by modulating IRE1 signaling. 

Many of the effects of aging have been attributed to oxidative stress [50]. However, unlike taurine deficiency, complex III is a primary source of mitochondrial ROS generation in aging. Because complex III targets ROS to the intermembrane space while taurine deficiency in young rodents or isolated cells is associated with matrix oxidative stress [9,32,39], oxidative stress is more widely distributed in the 12-month-old TauTKO heart than in the young TauTKO heart. These differences in ROS compartmentalization provide a logical explanation for the differences in UPR signaling between the older and younger TauTKO hearts. One of the UPR pathways that should be sensitive to these differences is IRE1, which is a determinant of caspase 12 activation state. The regulation of IRE1 signaling is extremely complicated, involving ROS, protein phosphorylation, CREB, TRAF2, Bcl-2 family of proteins, and a scaffold [51]. Nonetheless, the data support a role for ER stress and IRE1 signaling in the activation of caspase 12 in 12-month-old TauTKO hearts. These alterations likely contribute to the severity of the cardiomyopathy that develops in TauTKO mice.

## 5. Conclusions

The present study outlines a novel mechanism for initiation of taurine deficiency-mediated apoptosis (Figure 8). We propose that declines in mitochondrial taurine content lead to a decrease in the formation of the taurine conjugate, 5-taurinomethyluridine-tRNA (Leu-UUR), which in turn alters the binding of the anticodon of the tRNA to the UUG codon of mitochondrial mRNAs. Consequently, the biosynthesis of ND6, whose mRNA contains 8 UUG codons, significantly declines. Because ND6 serves as a subunit of complex I and facilitates assembly of the complex, activity of complex I also declines in taurine deficiency. The resulting disruption of electron flow through the respiratory chain leads to the diversion of electrons to oxygen, increasing the formation of superoxide and other damaging oxidants. Mitochondrial damage leads to increased membrane permeability, releasing cytochrome c that contributes to the formation of the apoptosome and the activation of caspases 9 and 3, the latter an effector caspase that promotes apoptosis.

In aging, oxidative stress initiates ER stress, which upon stimulation of the UPR pathways, activates caspase 12, an initiating caspase that further stimulates caspase 3 and apoptosis. The rise in cardiac apoptosis promotes the development of cardiomyopathy, increasing mortality in taurine deficiency. Thus, taurine is an important nutrient that is required for normal mitochondrial function. Hence, taurine deficiency reduces lifespan by promoting mitochondrial-dependent and ER stress-mediated apoptosis.
